# Life-threatening bleeding after mastectomy and deliverance by a single suture: A case-report

**DOI:** 10.1016/j.jpra.2025.03.024

**Published:** 2025-04-03

**Authors:** Anselm Tamminen

**Affiliations:** a Department of Plastic and General Surgery, Turku University Hospital, Kiinamyllynkatu 4-8, 20520 Turku, Finland; b Department of Surgery, Faculty of Medicine, University of Turku, Kiinamyllynkatu 10, 20520 Turku, Finland

**Keywords:** Mastectomy, Bleeding, Complications, Treatment

## Abstract

Postoperative bleeding after mastectomy is rarely a life-threatening complication. However, it can be fatal without treatment, particularly in patients taking antithrombotic medications. We present a case of 84-year-old woman with atrial fibrillation and apixaban medication, who developed breast cancer requiring mastectomy. The patient experienced severe post-operative bleeding on the fifth postoperative day, with first symptoms being disorientation, stomachache, and unstable hemodynamics. A contrast-enhanced computed tomography revealed an active bleeding site superficially in the skin flap. Unfortunately, the patient had to face a two-hour delay before emergency surgery could be performed and the on-call surgeon decided to attempt an unconventional treatment. The source of the bleeding was pinpointed using Doppler ultrasound in the emergency room, and the artery was occluded with a large needle and a figure-of-eight suture.

## Introduction

Mastectomy is one of the most common surgical procedures. Due to the aging population, and the increasing incidence of breast cancer, patients who take antithrombotic medications and require mastectomy are encountered with increasing frequency. Bleeding is one of the most common complications after mastectomy.^1^ While it most often occurs during the first 24 h after the surgery, it may present several days, or in rare cases, even weeks after the operation.^2^ At that time the patient has usually been discharged from the hospital, severing the potential consequences of the complication. Postoperative bleeding after mastectomy is seldom a life-threatening complication, but it can be fatal, especially in patients taking antithrombotic medications.^3^

In this article, we report an 84-year-old woman who took apixaban for atrial fibrillation and underwent mastectomy with axillary lymph node dissection for breast cancer. After uneventful surgery, the patient was successfully discharged. Later, on the fifth postoperative day, the patient suffered life-threatening bleeding, which was managed by the creative actions of the on-duty surgeon in emergency room, as the patient could not receive immediate emergency surgery.

## Case report

An 84-year-old woman had a history of atrial fibrillation, hypertension, coronary artery disease, hypothyroidism and type 2 diabetes. The patient also had a history of 50 years of smoking. Due to the atrial fibrillation, the patient took apixaban 5 mg once a day. The patient resided in a nursing home. The patient was diagnosed with multifocal ductal breast cancer and axillary metastases.

The patient underwent mastectomy with axillary lymph node dissection at a tertiary teaching hospital. Mastectomy was performed with a diathermy scalpel and the axillary lymph node dissection with an energy instrument. The operation itself was uneventful. Blood loss during surgery was 150 ml. Apixaban was discontinued on the day of the operation. There were no signs of complications postoperatively, the medication was resumed at the normal dose on the day following the operation. The patient was monitored on the ward for two days. The recovery began as usual, and the patient was discharged in good condition the third day after the surgery.

However, on the evening of the fifth postoperative day, the patient was found in her room in nursing home, disoriented and complaining of stomachache. An ambulance was called, and upon its arrival the patient presented with cold periphery, and the blood pressure was too low for successful measuring. The mastectomy site was recorded to be “neat”, raising no suspicion of surgical complications.

The patient was transferred to the hospital's emergency room (ER). During transportation, the patient received 500 ml of Ringer's solution and 6 mg of ephedrine. When arriving at the ER, the blood pressure was recorded at 70/40 mmHg. The patient was disoriented but conscious, answering the questions with one or two words. As she complained of severe stomachache, a contrast-enhanced computed tomography (CT) scan was performed upon arrival. At that time, the mastectomy site was recorded to be “slightly swollen and showing signs of a hematoma”.

**Figure 1:**
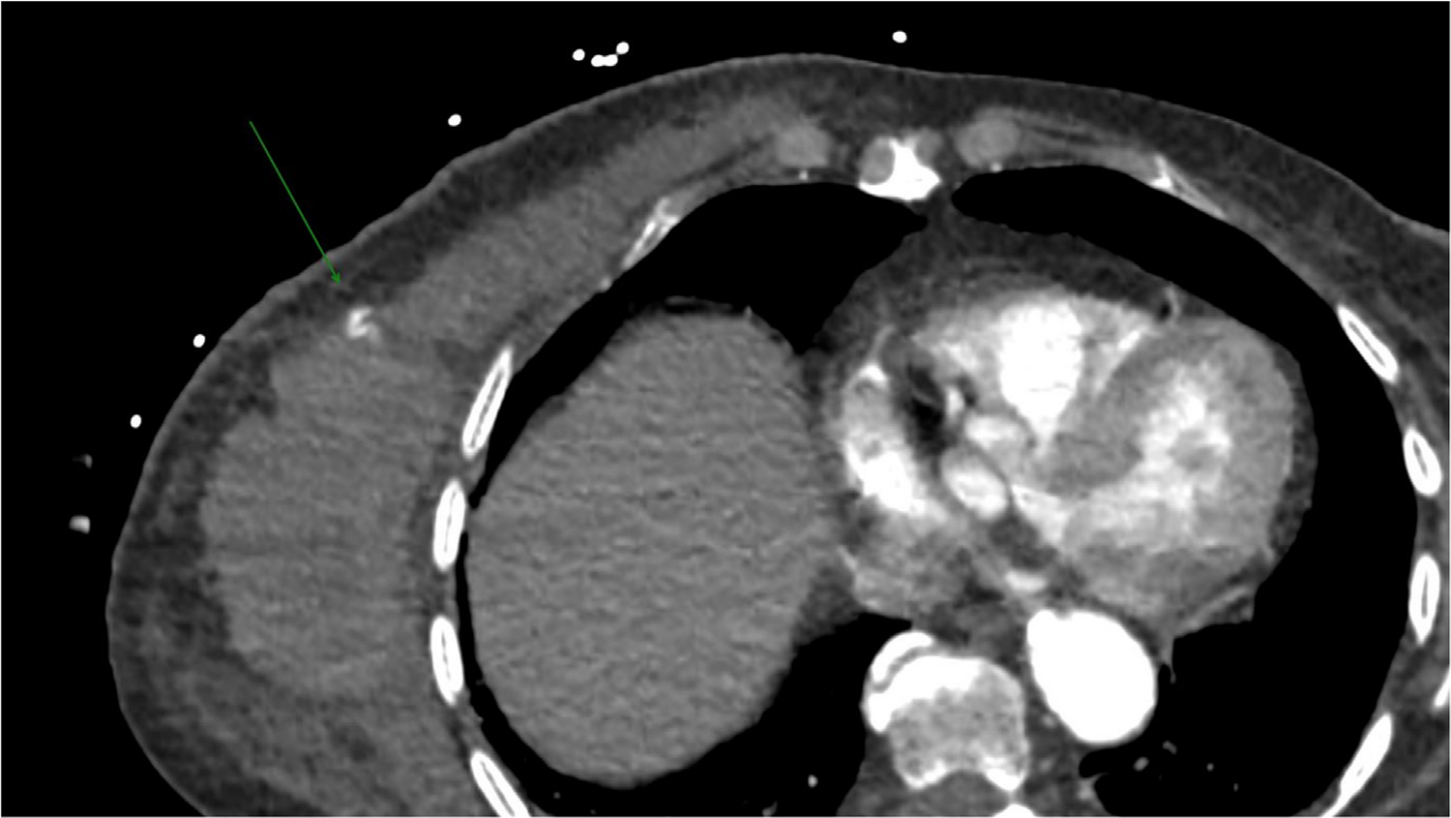
The computed tomography revealed an active bleeding from the superficial skin flap.

The CT scan revealed no abnormalities in the abdominal region, but it did show an extensive hematoma at the mastectomy site and active bleeding from an artery on the caudal skin flap (Figure 1).

As the patient arrived at the hospital late in the evening, the hospital had only one operating room prepared for surgery. Unfortunately, another critically ill patient had just been taken into the operating room. The surgeon about to start the operation estimated that it would take at least two hours before the mastectomy patient could undergo surgery she urgently required. While treatment options were being assessed by the surgeon and the anesthesiologist, the patient was prescribed 1 g of tranexamic acid, and the patient was given intravenous fluids to support hemodynamics.

At that point, the surgeon responsible for the ER patients contacted the radiologist on-duty and requested an ultrasound study to pinpoint the exact location of the bleeding artery. Using Doppler mode, the artery still actively bleeding was identified. The surgeon decided to experiment placing a figure-eight suture with the largest available needle at this site. The ultrasound examination was repeated, showing no further signs of active bleeding. In co-operation with the anesthesiologist, the patient was gradually stabilized, and after approximately two hours of waiting, the patient could finally be taken into the operating room. In the operation, the surgeon removed 1700 ml of blood. No active bleeding site was detected. The patient eventually recovered from this event, but unfortunately, was later diagnosed with distant metastases. Palliative care was initiated, and the patient soon passed away.

## Discussion

Bleeding complications after mastectomy are rarely severe but can be life-threatening, especially in patients taking antithrombotic medications. However, discontinuing antithrombotic medication can also lead to complications, such as stroke and pulmonary embolism, during the postoperative period especially in patients with atrial fibrillation.^4^ Therefore, the optimal perioperative protocol for antithrombotic medications remains uncertain. It has been shown that utilizing ultrasound instruments may be used in patients on concurrent antithrombotic medication without an increased risk of bleeding complications.^5^ Although ultrasound instrument is conventionally utilized for mastectomy at our institute, the operation in this patient was performed with a diathermy scalpel, which has been associated with a higher risk of bleeding complications compared to ultrasonic instruments.^2^ It may be speculated whether this incident could have been avoided if ultrasonic instrument was used in the surgery.

The diagnosis of bleeding complications after mastectomy may be challenging, as the surgical cavity is large, and a substantial amount of blood may be spread to the area before it becomes outwardly apparent. Recognizing the risk for obscure surgical complications is important when evaluating postoperative deterioration of the patient's condition. As bleeding itself does not necessarily cause symptoms, an entirely incorrect diagnosis may be suspected in the beginning. In the present case, the patient complained of stomachache, although no pathology in abdominal area was present. More than an hour passed after the patient was found disoriented in her room, before the hematoma became outwardly apparent. Retrospectively, the most plausible explanation for the symptom is mesenterial ischemia, caused by hypovolemia and hemodynamic instability, with similar phenomenon sometimes observed in patients with cardiac arrest.^6^

Another matter to consider is the selection for appropriate surgery for patients with multiple underlying conditions. Mastectomy carries a higher risk of complications compared to breast-conserving surgery.^7^ In cases like this one, where the patient has a limited life expectancy, one might consider whether breast-conserving therapy, in some cases possibly even without adjuvant therapies, should be considered the best option for the patient.

## Conclusion

Postoperative bleeding after a mastectomy can be a life-threatening complication. The diagnostics of bleeding complications after mastectomy can be challenging, because the potential space for internal bleeding after a mastectomy is large, and the signs of the hematoma become evident only when the amount of bleeding is substantially large. Sometimes unconventional measures may help save the patient's life.

## Funding

This study has been supported by a grant from the Cancer Society of Southwest Finland. The funder had no role in study design, data collection and analysis, decision to publish, or preparation of the manuscript.

## Declaration of competing interest

The authors declare that there is no conflict of interest.
